# Study of the Microscopic Mechanism of Natural Rubber (Cis-1, 4-Polyisoprene, NR)/Polyethylene (PE) Modified Asphalt from the Perspective of Simulation

**DOI:** 10.3390/polym14194087

**Published:** 2022-09-29

**Authors:** Yujing Chen, Kui Hu, Caihua Yu, Dongdong Yuan, Xiaoyi Ban

**Affiliations:** 1 School of Highway, Chang’an University, Xi’an 710064, China; 2 College of Civil Engineering, Henan University of Technology, Zhengzhou 450001, China; 3 Department of Structural Engineering, College of Civil Engineering, Tongji University, Shanghai 200092, China

**Keywords:** asphalt pavements, modified asphalt, functional polymer, cis-1, 4-polyisoprene, polyethylene, molecular dynamics, microscopic mechanism

## Abstract

This paper aims to study the interaction mechanism of waste tire/plastic modified asphalt from the microscopic perspective of molecules. Based on BIOVIA Materials Studio, a classic four-component asphalt model consisting of asphaltene (C_149_H_177_N_3_O_2_S_2_), resin (C_59_H_85_NOS), aromatic (C46H50S), and saturate (C_22_H_46_) was constructed. Waste tires are represented by natural rubber (NR), which uses cis-1, 4-polyisoprene as a repeating unit. In contrast, waste plastics are characterized by polyethylene (PE), whose optimum degree of polymerization is determined by the difference in solubility parameters. Then, the above molecular models are changed to a stable equilibrium state through the molecular dynamics process. Finally, the interaction process is analyzed and inferred using the indexes of radial distribution function, diffusion coefficient, and concentration distribution; further, the interaction mechanism is revealed. The results show that the optimal degree of polymerization of PE is 12, so the solubility parameter between PE and NR-modified asphalt is the lowest at 0.14 (J/cm^3^) ^1/2^. These models are in agreement with the characteristics of amorphous materials with the structures ordered in the short-range and long-range disordered. For NR-modified asphalt, the saturate moves fastest, and its diffusion coefficient reaches 0.0201, followed by that of the aromatic (0.0039). However, the molecule of NR ranks the slowest in the NR-modified asphalt. After the addition of PE, the diffusion coefficient of resin increased most significantly from 0.0020 to 0.0127. NR, PE, and asphaltene have a particular attraction with the lightweight components, thus changing to a more stable spatial structure. Therefore, using NR and PE-modified asphalt can change the interaction between asphalt molecules to form a more stable system. This method not only reduces the large waste disposal task but also provides a reference for the application of polymer materials in modified asphalt.

## 1. Introduction

Petroleum asphalt is an essential raw material in road engineering, especially in asphalt pavements, which are used widely. At present, petroleum is gradually becoming exhausted, so asphalt, a by-product of petroleum, will also face the problem of exhaustion. Against this background, it is indispensable and crucial to lower the consumption of asphalt in road engineering and decrease dependence on petroleum. In addition, the deteriorating environment and traffic conditions put higher requirements on the performance of asphalt pavements. Therefore, the modification of asphalt to reduce cost and improve performance is one of the leading research directions for researchers, which is very significant. The use of modified asphalt has recently increased, and its application scope and applications are becoming broader [1,2,3,4,5].

**Figure 1:**
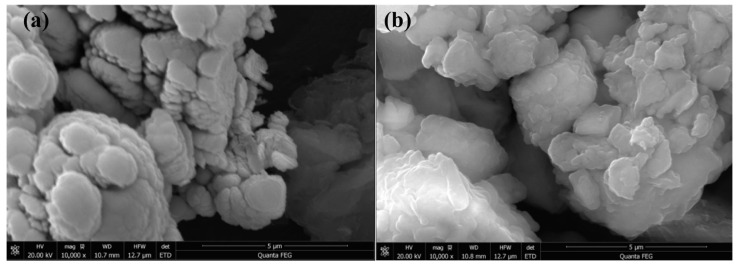
The SEM images of (**a**) BT and (**b**) OBT.

There are many kinds of materials that can be used as modifiers, and the current research mainly includes the following aspects. Some researchers use biomaterials as modifiers to prepare bio-asphalt [6,7,8], such as beans [9], waste cooking oil [10,11], biochar [12,13], and so on. Forestry resources and animal excreta are liquefied and separated to prepare modifiers. In addition, in the process of biomass raw materials processing to produce bio-based materials (furfural, furfuryl alcohol, dimethylfuran, biodiesel, etc.), the remaining polymer carbohydrates can also be used to create bio-asphalt. Some researchers use soil as a modifier, such as nano clay [14], diatomite [15,16], organically modified bentonite [17,18] and so on. Take bentonite as an example. Its electron microscope diagram is shown in Figure 1, where (a) represents the original bentonite and (b) represents the nano-bentonite, which shows a clear layered structure and good continuity. Bentonite itself belongs to the octahedral structure. If added to the asphalt, it can fill the pores between the micelle and micelle and further increase the anti-skid resistance of asphalt. Fibers are also adopted as modifiers, such as polyester [19,20], lignin [21,22], basalt fibers [23,24] and so on [25]. This kind of modifier can enhance the cohesion between aggregate particles so that the asphalt pavement can adapt to high wear requirements, increased skid resistance, and other sections. Guo [26,27], Zheng [28], and some other researchers [29,30] used waste tires as raw materials to prepare rubber-modified asphalt and conducted research. Some researchers use plastics as modifiers, such as polyethylene, polyurethane, polypropylene, etc. [4,31,32]. Some nano-modifiers are carbon nanotubes, graphene, and other nanomaterials, but the cost is high.

It should be noted that as asphalt modifiers, waste tires and plastics have a significant negative influence on the ecological environment [33]. Waste tires and plastics are non-biodegradable substances, which brings difficulties to waste management [27]. If incinerated, a lot of smoke and other harmful substances will be produced, which will significantly impact the air, soil, rivers, and other environments. However, waste plastics and rubber have good reuse value as asphalt modifiers and can improve asphalt performance [28]. Generally speaking, plastic polymers can significantly enhance asphalt performance at high temperatures. In contrast, rubber polymers can upgrade the deformation resistance of asphalt at low temperatures, such as styrene-butadiene rubber (SBR). Therefore, the use of the two as asphalt modifiers simultaneously becomes one of the best choices for the recycling and reuse of waste material. At present, only one kind of modified agent is doped separately. Still, there are few studies on adding two types of waste materials simultaneously. Hence, it is not easy to give full play to the synergistic effect of the two and improve the high- and low-temperature performance of asphalt simultaneously.

It is a much more cost-effective and scientific to perform research using molecular dynamics. The traditional test has a long test cycle and a high cost. In addition, many conventional experiments only describe and compare the experimental results, which fails to explain some empirical phenomena in depth. Molecular dynamics, on the other hand, refers to the study of microscopic phenomena from the perspective of molecules or atoms. Currently, many researchers are studying the molecular dynamics of asphalt, such as the modification mechanism of modifiers [34]. For example, Hu et al. [35] studied the interaction mechanism between styrene-butadiene-styrene (SBS) and asphalt to explain the formation and separation mechanism of an SBS-modified asphalt two-phase structure from a microscopic perspective.

The purpose of this paper is to study the molecular interaction mechanism after two modifiers simultaneously using molecular dynamics. In this paper, the original asphalt and rubber-modified asphalt models were built from the perspective of molecular dynamics. Then, plastic was added to create a plastic/rubber asphalt model, and the influence of plastic molecules on rubber asphalt was analyzed. Among the models, the classical four-component molecular model was taken to represent asphalt, and rubber is expressed by natural rubber molecules composed of polyisoprene. Additionally, plastics are represented by polyethylene molecules, the optimal polymerization degree of which is measured by solubility parameters. After the molecular dynamics process, all the above models reach a state of equilibrium and stability. The radial distribution function (RDF) was used to verify the molecular model, and the mean square displacement (MSD) and diffusion coefficient were used to study the diffusion of molecules in modified asphalt. Finally, concentration distribution was taken to study the spatial distribution of different molecules.

## 2. Simulation

### 2.1. Model of Asphalt

Bitumen is a highly complex mixture of hydrocarbons of different molecular weights and their derivatives of non-metallic components (oxygen, nitrogen, sulfur, etc.). Petroleum bitumen, a vital type of bitumen, is widely used in road engineering. From a scientific research perspective, the parts of petroleum asphalt with some common characteristics, such as similar chemical composition and physical properties, are divided into the same group, called components. The American Society for Testing and Materials (ASTM) D4124-09 has proposed and recommended a four-component analysis method. This method divides petroleum asphalt into asphaltene, aromatic, resin, and saturate.

**Figure 2:**
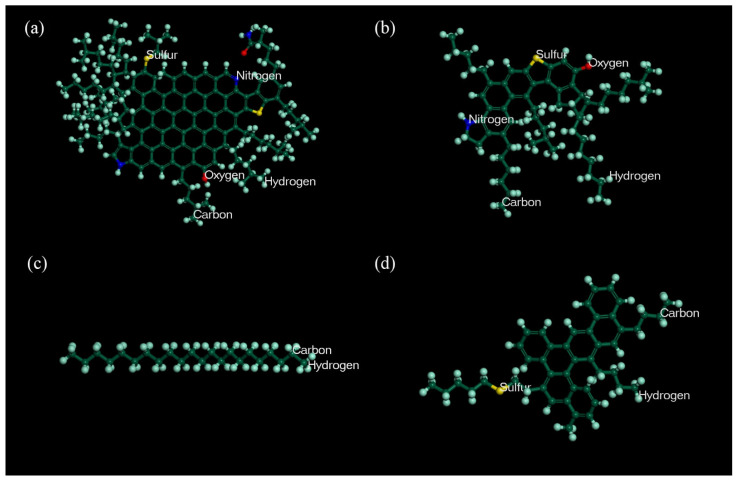
Models of representative molecules in bitumen. (**a**) asphaltene; (**b**) resin; (**c**) saturate; (**d**) aromatic.

The simplified model adopted in this study consists of four representative molecules, and this method has been widely used because of its simplicity, convenience, and conformity with the actual situation [36]. The four representative molecules are asphaltene (C_149_H_177_N_3_O_2_S_2_), resin (C59H85NOS), aromatic (C46H50S), and saturate (C_22_H_46_), as shown in Figure 2. The proportion of the four representative molecules in this model is calculated according to the mass ratio and molecular weight of each component in asphalt, in line with previous studies [36,37], as shown in Table 1.

### 2.2. Models of Rubber

Rubber powder mainly comes from waste tires, and its composition is complicated. The three most common types of rubber are NR (natural rubber), BR (cis-polybutadiene rubber), and SBR (styrene-butadiene rubber). Since natural rubber is the most widely used, it was selected as the research object in this study.

**Figure 3:**
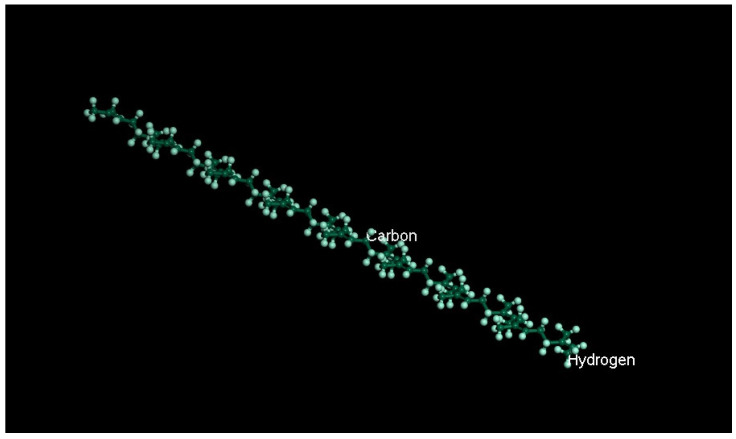
The molecular chain for NR.

Natural rubber mainly comprises cis-1, 4-polyisoprene, accounting for more than 97% and 2–3% of the 3- and 4-bonded structure. Therefore, when building the molecular model of rubber, NR was regarded as a homopolymer, which was polymerized from the isoprene mentioned above. The relative molecular weight range of NRs is relatively wide, generally between 30,000 and 30 million, because of the different degrees of polymerization. The degree of polymerization affects the results of the experiments and leads to different simulation costs, so a proper selection is needed. Referring to previous studies [29,38], the degree of polymerization selected in this paper is 16, and the number of repeating units is set at 16. The molecular chain for NR is shown in Figure 3.

### 2.3. Models of PE

Polyethylene, short for PE, is a thermoplastic resin prepared by the polymerization of ethylene, including ethylene and a small amount of α-olefin copolymer. The degree of polymerization, a key factor, affects the molecular weight and the amount of simulation, and it is also closely related to the solubility parameters. The independent variables of the polymerization degree for polyethylene used in this article are 6, 12, 18, 24, 30, 36, 42, 48, 54, 60, 66, 72, 78, 84, 90, 96, 102, 108, 114, 120, and there are 20 different degrees of polymerization in total. Firstly, the degree of polymerization is closely related to the amount of simulation calculations because the degree of polymerization is a direct factor affecting the molecular weight; the greater the degree of polymerization, and the greater the molecular weight, the larger the amount of simulation calculations. Secondly, the solubility parameters of polyethylene with different degrees of polymerization are different. If the difference in the solubility parameters is less than 1.3~2.1 (J/cm^3^) ^1/2^, the two materials are compatible. The smaller the difference is, the more easily the two are compatible. Therefore, in this paper, the solubility parameter difference is selected to evaluate the solubilization effect of PE on rubber-modified asphalt.

### 2.4. Models of the Mixture

The simulation software used in this study is the BIOVIA Materials Studio, an integrated computer simulation platform involving quantum mechanics, molecular dynamics, and mesoscopic dynamics. Its function is to reveal the interaction mechanism of substances or predict the properties of materials from the microscopic perspective of molecules or atoms; therefore, it is widely used in various research fields.

The basic principles of molecular dynamics simulation were introduced. A system with N particles and the description of classical mechanics depends on 3N degrees of freedom $\left(q,p\right)\;\equiv \left\{q_{i,}p_{i}|i=1\;\left(1\right)\;N\right\}$. The position $q\equiv \left\{q_{i}|i=1\;\left(1\right)\;N\right\}$, momentum $P\equiv \left\{p_{i}|i=1\left(1\right)N\right\}.\;q_{i}$ is the position of the particle $No_{.}\;i$ and $p_{i}$ is the linear momentum. (q,p) determines the system’s state, and each microscopic state is a phase point in phase space.

In molecular dynamics studies, the laws of Newtonian mechanics are usually adopted, as shown in the related literature [39].

(1) $$\frac{dv_{i}}{dt}=\frac{F_{i}\;\left(t\right)\;}{m_{i}}=-\frac{1}{m_{i}}\nabla_{i}U\;\;\;\forall i=1\left(1\right)N$$

(2) $$\;\frac{dr_{i}\;\left(t\right)\;}{dt}=v_{i}\;\left(t\right)\;\;\;\forall i=1\left(1\right)N$$

where, $r_{i}$ and $v_{i}$ are the position and velocity of particle No. i, respectively. The bond action, similarly to the quantum effect in the above equation, is reflected in the potential energy U. The potential energy U, which depends on the position of all of the nuclei, can be calculated precisely by quantum chemistry or approximated by force-field methods.

Formula (1) and (2) are a set of 3N differential equations, which should be solved by the numerical integration method of differential equations. Therefore, the position $\left\{r_{i}\left(t\right)\right\}$ and velocity $\left\{v_{i}\left(t\right)\right\}$ of the system at each moment can be solved by the integration algorithm. In this case, the trajectory of all the nuclei of the system is given, that is, the time evolution of the system. Then, the change of all system properties with time can be obtained.

COMPASS is a powerful force field that supports the atomic level simulation of condensed matter materials, short for “Condensed-Phase Optimized Molecular Potential for Atomistic Simulation Study.” It is the first ab initio force field parameterized and verified by condensed matter properties and various ab initio and empirical data of isolated molecules. Therefore, the structure, conformation, vibration, and thermophysical properties of different molecules in isolated or condensed systems could be accurately predicted for a wide range of temperatures and pressures by this field [40,41,42,43,44].

The specific steps of building a stable model are as follows.

**Figure 4:**
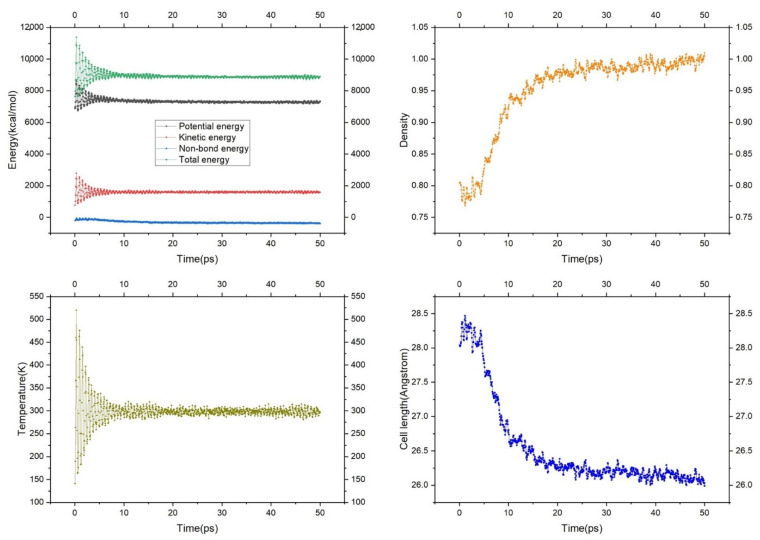
Dynamic equilibrium process.

1. The establishment of initial polymer model.
2. Geometric optimization.
3. The simulation of annealing.
4. In this procedure, the pressure (1.01 × 10^−4^ Gpa) and temperature of the system remain constant [45].
5. NVT+NPT dynamics simulation.
6. At ambient temperature and standard atmospheric pressure, 50 ps of MD simulation was performed to bring the model closer to its natural state [46,47]. Taking the original asphalt model as an example, after the above steps were performed, parameters such as the energy, density, and others for the system reached a stable state, as shown in Figure 4. For example, the density of the original asphalt model increased and stabilized at about 1.0, which coincided with the actual situation.
7. The calculation and analysis of parameters.
8. After the molecular dynamic simulation, parameters related to this study were analyzed and calculated, such as the solubility parameters and radial distribution function, etc., according to the stable model generated at the end of the simulation process.

**Figure 5:**
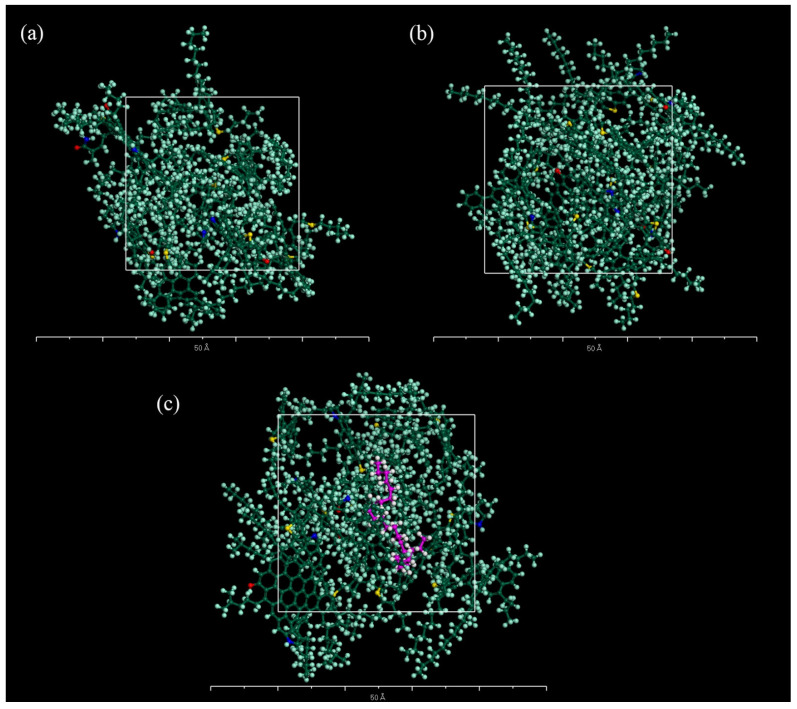
Models (**a**) original asphalt; (**b**) NR-modified asphalt; (**c**) PE/NR-modified asphalt.

The specific composition of the two modified asphalt models, including NR-modified asphalt and PE-modified asphalt, is shown in Table 2 and Table 3. Both the original asphalt model and the modified asphalt model obey the above steps. In the NR-modified asphalt, one NR molecule was included, and its content was 9.28%. The original asphalt model and NR-modified asphalt model in an equilibrium state are shown in Figure 5.

In PE/NR-modified asphalt, the degree of polymerization of the PE molecule should first be determined. In this study, different molecular models were constructed for PE molecules with varying degrees of polymerization, and the kinetic simulation process was performed according to the steps mentioned above. The equilibrium model was finally obtained, as shown in Figure 5.

## 3. Results and Discussion

### 3.1. Solubility Parameters

**Figure 6:**
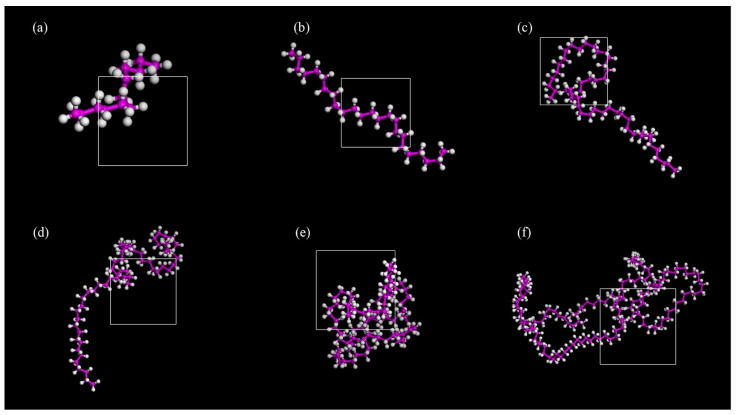
PE molecular model with different degrees of polymerization (**a**) 6 (**b**) 12 (**c**) 24 (**d**) 30 (**e**) 42 (**f**) 78.

Molecular models of PE with different degrees of polymerization are shown in Figure 6. The solubility parameters were used to evaluate the compatibility between polyethylene with different degrees of polymerization and NR-modified asphalt. If the solubility parameters for two polymer materials become closer, the blending effect is better [42]. In this study, solubility parameters were adopted to evaluate the compatibility between different polymers, primarily to determine the degree of polymerization of PE molecules. Its value is the square root of the cohesive energy density, as shown in the following Formula (3):

(3) $$\delta =\sqrt{CED}=\sqrt{\frac{∆E}{V}}$$

where ΔE represents cohesive energy and V represents volume.

If the action of the hydrogen bond is ignored, the intermolecular interaction force mainly consists of a van der Waals force and electrostatic force; the CED is composed of the van der Waals cohesive energy density ($CED_{v}$) and electrostatic cohesive energy density ($CED_{e}$).

**Figure 7:**
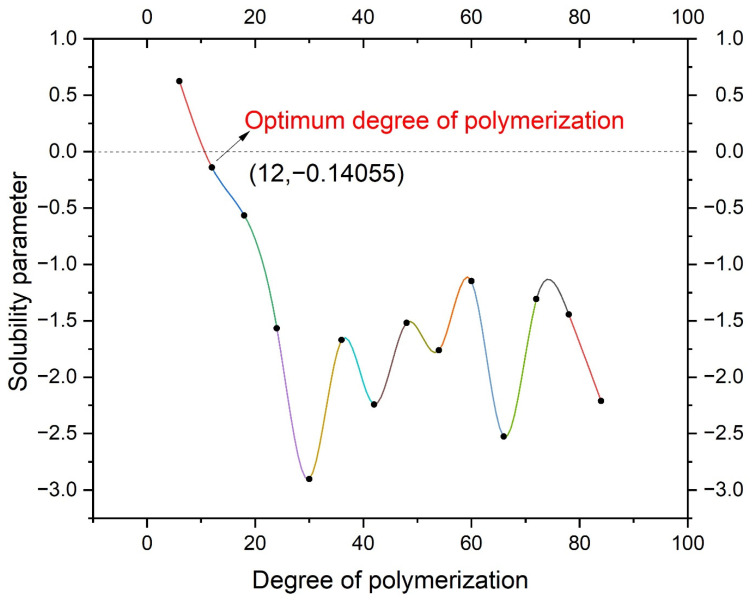
The difference in the solubility parameter.

Then, the related cohesive energy index is obtained and used to estimate the solubility parameter. The solubility parameters of the PE additives with individual degrees of polymerization are shown in Table 4, while that of the NR-modified asphalt is 17.23. The difference between the two is shown in Figure 7. According to the principle of similarity-compatibility, when the degree of polymerization is 12, the difference between the two is the smallest, which is 0.14 < 1.3~2.1 (J/cm^3^) ^1/2^. Therefore, the optimum polymerization degree of the PE molecule in this paper is 12.

A PE molecular chain with a polymerization degree of 12 was adopted to establish a modified asphalt model. After the steps of dynamics simulation mentioned above were performed, the balanced model of PE/NR modified asphalt was finally obtained for subsequent analysis and calculation.

### 3.2. Radial Distribution Function

RDF, an essential indicator in molecular dynamics, can be employed to verify the correctness of molecular models.

The radial distribution function (RDF) can be applied to analyze the interaction and microscopic distribution of particles [39,43,44]. The radial distribution function is the spatial probability distribution of particle B approaching the center of particle A, which is in the r to $r+d_{r}$ shell. It can also be understood as the ratio of the number density of particle B to the mean density of particle B in this region.

(4) $$g\left(r\right)=\frac{dN}{\rho V}$$

dN is the number of particles B in the shell r to $r+d_{r}$; ρ is the numerical density of particle B.

V is the volume of the spherical shell, centered on atom A, with the radius R and the thickness $d_{r}$. The formula is as follows:

(5) $$V=\frac{4}{3}\pi \;\left(r+dr\right){\;}^{3}-\frac{4}{3}\pi r^{3}=4\pi r^{2}dr+4\pi rdr^{2}+\frac{4}{3}\pi dr^{3}\approx 4\pi r^{2}dr$$

For the RDF of amorphous crystals, there are a few peaks with different heights and sharpness at close distances, but the peak height decreases rapidly with an increase in space. At long distances, the radial distribution function tends to be evenly distributed, g (r) = 1.

The RDF of the crystal still maintains a sharp peak shape at a long distance, indicating that the crystal has a long-range ordered structure, whereas the amorphous crystal has a short-range ordered structure and a long-range disorder structure.

### 3.3. Diffusion Coefficient

The diffusion coefficient can represent the molecular movement rate and is employed to study the changes of diverse molecular movements in NR-modified asphalt systems with and without PE. According to Einstein’s law [48], if the simulation time is long, the slope of the curve of mean square displacement (MSD) to time is six times the diffusion coefficient; thus, the diffusion coefficient of particles can be gained, as shown in formula (6). In molecular dynamics calculation, molecules move continuously from the initial position, and the position of each moment is different. If r (t) represents the position of particle i at time t, MSD represents the average square of displacement. The formula of the diffusion coefficient is shown in formula (6).

(6) $$D=\underset{t\to \infty }{\lim}\frac{MSD\;\left(t\right)\;}{6t}=\underset{t\to \infty }{\lim}\frac{{\left|r\left(t\right)-r\left(0\right)\right|}^{2}}{6t}$$

**Figure 8:**
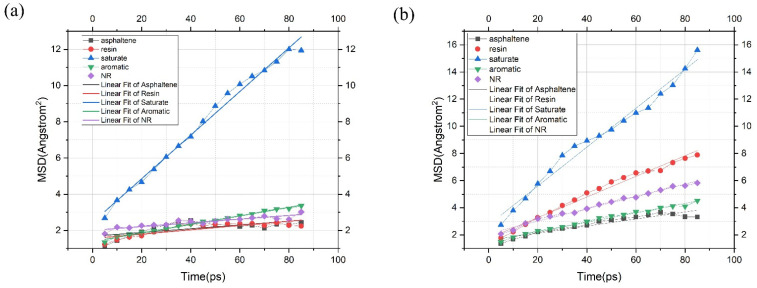
*MSD* for modified asphalt (**a**) NR-modified asphalt; (**b**) PE/NR-modified asphalt.

**Figure 9:**
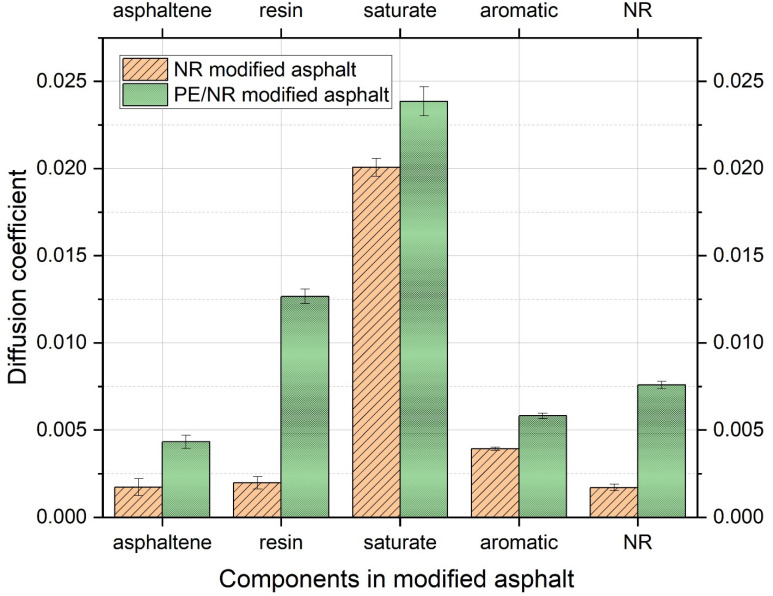
Diffusion coefficient of each component in NR modified asphalt and PE/NR modified asphalt.

To analyze the movement of various molecules in NR-modified asphalt, the MSD results of different molecules were obtained through molecular dynamics calculation, as shown in Figure 8. According to the functional relationship between the diffusion coefficient and MSD, the diffusion coefficient is obtained, as shown in Figure 9. The analysis shows that the saturate has the fastest movement speed, which is much higher than other types of molecules, and its diffusion coefficient reaches 0.0201. This phenomenon is most likely related to its negligible molecular weight. Aromatic ranks second, at about 0.0039.

In addition, the molecular movement velocity of asphaltene, resin, and rubber are roughly similar, showing that their diffusion coefficients are about 0.0018, likely connected with the higher molecular weight and unique shapes to some extent.

For the NR-modified asphalt system, the diffusion coefficient of NR is the lowest. More specifically, the long-chain shape of NR and its poor compatibility with the polar components of asphalt may be the main reasons restricting the movement of NR. Therefore, NR is the slowest molecule in the NR-modified asphalt system, with a diffusion coefficient of 0.00171. However, NR attracts non-polar components in asphalt (aromatic and saturate), which results in a more aromatic and saturated distribution around NR molecules. Some light components are attracted by NR molecules and transferred to the vicinity of NR molecules, so it could be noted that the light components have the highest diffusion coefficients, which are 0.0201 and 0.0039, respectively, in NR-modified asphalt.

As shown in Figure 9, the movement of molecules changed significantly due to the addition of PE. The diffusion coefficient of resin molecules increased dramatically from 0.0020 to 0.0127. This may be because there is a specific force between the PE and resin, thus accelerating the movement of resin molecules. According to the colloid theory for asphalt, resins attach molecules in asphalt to form micelles. In this study, the transfer of resin molecules caused the asphaltene molecules to lose part of the constraint, resulting in a slight increase in the diffusion coefficient of asphaltene from 0.00173 to 0.0043. Because of the rearrangement and combination of the dispersed phase, it is inevitable to promote the corresponding transfer of light components, including saturate and aromatic, that is, the corresponding increase of diffusion coefficient; for example, the diffusion coefficient of saturate increases from 0.0201 to 0.0239, and that of the aromatic component increases from 0.0039 to 0.0058.

In the statistical data analysis, the Pearson correlation coefficient is adopted to reflect the linear correlation of two random variables. For NR-modified asphalt and PE/NR-modified asphalt, MSD increased with time. Linear fitting was performed, and the Pearson correlation coefficient was analyzed. For NR-modified asphalt, the Pearson correlation coefficient of asphaltene and resin was 0.67805 and 0.82105, respectively, as shown in Table 5. Meanwhile, the PE/ NR modified asphalt coefficient is above 90%, 0.94514, and 0.99194, respectively, as shown in Table 6.

### 3.4. Concentration Distribution

Concentration distribution, an important indicator, can show how concentration varies with position in a region at a given time. This parameter can be acquired through the Forcite Analysis module in Materials Studio. The spatial distribution of each molecule in the NR modified asphalt in three different directions (100,010,001) and the spatial distribution of each molecule in the PE/NR modified asphalt were evaluated, and the interaction between each molecule was further analyzed and predicted. In the concentration distribution curve of a molecule, the peak represents the location at which the molecule is most abundant.

**Figure 10:**
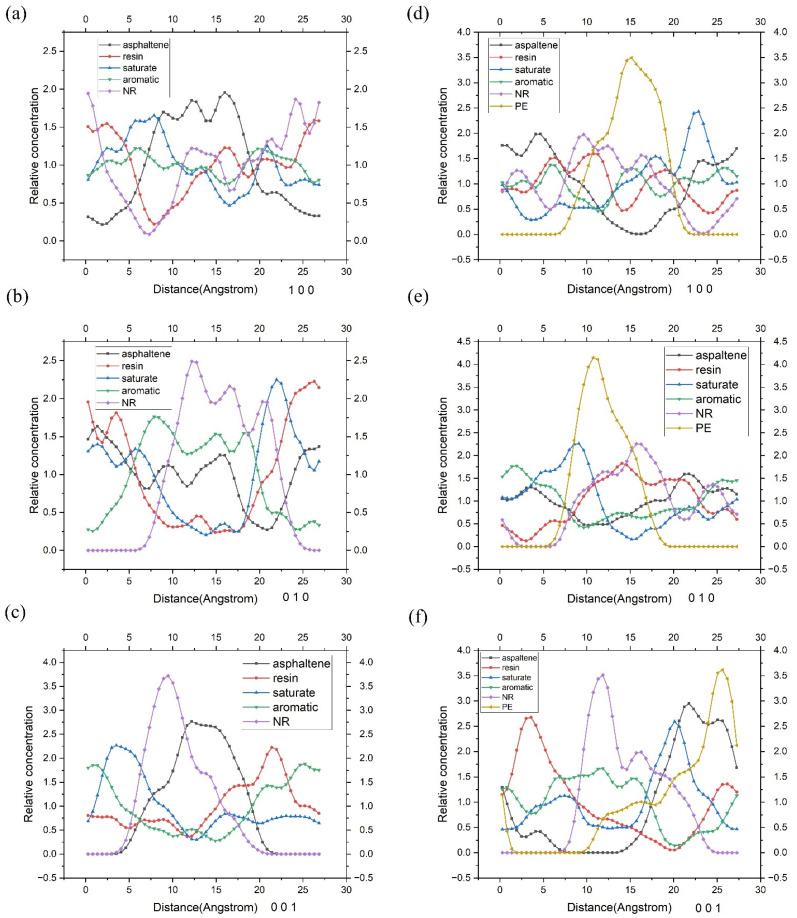
Concentration distribution of each component for NR-modified asphalt and PE/NR-modified asphalt. (The relative concentration of NR-modified asphalt in different directions, including (**a**) 1 0 0, (**b**) 0 1 0, and (**c**) 0 0 1; The relative concentration of PE/NR-modified asphalt in different directions includes (**d**) 1 0 0, (**e**) 0 1 0, and (**f**) 0 0 1).

As shown in Figure 10a–c, the four components of asphalt basically follow the colloid structure theory to form a blend with an ordered arrangement. After careful analysis, the following specific rules can be found. Firstly, it might be suggested that there is adsorption between the asphaltene, NR molecules, and the light components. Specifically, the concentration curve peak of asphaltene is surrounded by the concentration curve peak of saturate because the saturate is adsorbed around by asphaltene, and the mutual attraction between the two is considerable. Again, it is surrounded by a peak of aromatic. Both indicate adsorption between the asphaltene and light components. At the same time, the concentration curve peak of NR was also surrounded by the concentration curve peak of the light component, indicating that the light component was also adsorbed around the NR molecule. Second, the peak of the NR concentration curve is far away from that of resin, as shown in Figure 10b, which may be closely related to the incompatibility between the NR and resin, thus preventing them from coexisting in the same position. Similarly, the same rule can be found in the relationship between NR and asphaltene, which is more pronounced. As shown in Figure 10a, the concentration curves of the two have apparent peaks, and the distance between the two peaks is greater, indicating that there is probably a more vigorous competition for light components and incompatibility between NR and asphaltene. This is consistent with a previous study [49]. NR has the highest binding strength to aromatic and saturate, followed by resins, and has the lowest binding capacity to asphaltenes.

Figure 10d–f shows the concentration distribution curve of PE/ NR-modified asphalt. It could be predicted from this group diagram that the addition of PE changes the colloid structure composition of asphalt. As shown in Figure 10d, both the PE concentration curve and that of asphaltene have evident peak values, and the peaks of them are far away. It could be the case that the PE molecule has a specific repulsive force with asphaltene. It can also be seen from Figure 10d that the concentration curve of the PE molecule overlaps with that of the saturate to a certain extent, and the saturation fraction is found near the periphery of the peak, which might be related to the intense attraction between the two. It is likely that the adsorption of the saturate could make the PE molecule move and form a more stable system. Therefore, PE molecules tend to further promote the formation of a new balanced and stable system by changing the interaction between the original system.

## 4. Conclusions

In this paper, two types of polymer materials, PE (polyethylene) and NR (cis-1, 4-polyisoprene), were used as modifiers to study the interaction mechanism in the two modified asphalts. Specifically, the interaction of molecules in the original asphalt and NR-modified asphalt was analyzed before and after the addition of PE. The change of the interaction can help the modified asphalt to become a more stable in structure and thus have better storage performance, which has a reference value for changing the molecular agglomeration phenomenon. On the other hand, the reuse of PE and NR, two typical wastes, is of great significance for the protection of the ecological environment.

The specific conclusions are as follows.

1. The optimal degree of polymerization of PE in this study model is 12. In this case, the solubility parameter between PE and NR-modified asphalt is the smallest at 0.14 < 1.3~2.1 (J/cm^3^) ^1/2^.
2. The three models in the paper are typical amorphous substances with structures of short-range order and long-range disorder. According to the RDF diagram, when r = 1.11, the g (r) functions for the three models all have sharp peaks, which are 11.76, 11.74, and 11.8, respectively.
3. In NR-modified asphalt, the diffusion coefficient of saturate is the largest, at far higher than that of other types of molecules, and its value reaches 0.0201. In addition, the molecular velocity of asphaltenes, resins, and rubbers are about the same, showing that their diffusion coefficients are about 0.0018. NR is the slowest molecule.
4. In PE/NR-modified asphalt, the movement of molecules changed significantly. The diffusion coefficient of resin molecules increased considerably from 0.0020 to 0.0127. The most noticeable feature is a specific adsorption phenomenon between the PE molecule and light components.

## Figures and Tables

**Table 1:** Specific information about the asphalt model.

Components	Molecular Formula	Number Ratio	Relative Molecular Mass	Mass Fraction (%)	NumAtoms
Asphaltene	C_149_H_177_N_3_O_2_S_2_	1	2106.19	19.734	333
Resin	C_59_H_85_NOS	3	856.395	24.071	147
Saturate	C_22_H_46_	5	310.610	14.551	68
Aromatic	C_46_H_50_S	7	634.966	41.645	97

**Table 2:** NR-modified asphalt model.

Serial Number	Representative Molecule	Chemical Composition	Molecule Number	Relative Molecular Mass	Mass Fraction
1	Asphaltene	C_149_H_177_N_3_O_2_S_2_	1	2106.190	17.90%
2	Resin	C_59_H_85_NOS	3	856.395	21.84%
3	Saturate	C_22_H_46_	5	310.610	13.20%
4	Aromatic	C_46_H_50_S	7	634.966	37.78%
5	NR	C_80_ H_130_	1	1091.920	9.28%

**Table 3:** PE/NR-modified asphalt model.

Serial Number	Representative Molecule	Chemical Composition	Molecule Number	Relative Molecular Mass	Mass Fraction
1	Asphaltene	C_149_H_177_N_3_O_2_S_2_	1	2106.190	17.40%
2	Resin	C_59_H_85_NOS	3	856.395	21.23%
3	Saturate	C_22_H_46_	5	310.610	12.83%
4	Aromatic	C_46_H_50_S	7	634.966	36.72%
5	NR	C_80_ H_130_	1	1091.920	9.02%
6	PE	C_24_H_50_	1	338.664	2.80%

**Table 4:** Solubility parameters of different PE molecules.

Number	1	2	3	4	5	6	7	8	9	10	11	12	13	14
Polymerization degree	6	12	18	24	30	36	42	48	54	60	66	72	78	84
Solubility parameter	17.85	17.09	16.66	15.66	14.33	15.56	14.99	15.71	15.47	16.08	14.70	15.92	15.79	15.02

**Table 5:** Data analysis of MSD for NR-modified asphalt.

Equation	y=a+b∗x				
Plot	asphaltene	resin	saturate	aromatic	NR
Weight	No Weighting				
Intercept	1.68015 ± 0.14844	1.55818 ± 0.1093	2.44376 ± 0.15638	1.38694 ± 0.02799	2.007 ± 0.06071
Slope	0.01035 ± 0.0029	0.01188 ± 0.00213	0.12047 ± 0.00305	0.02361 ± 5.46305E-4	0.01028 ± 0.00118
Residual Sum of Squares	1.28424	0.69631	1.42537	0.04566	0.21482
Pearson’s r	0.67805	0.82105	0.99522	0.99601	0.91308
R-Square (COD)	0.45975	0.67412	0.99046	0.99204	0.83371
Adj. R-Square	0.42373	0.65239	0.98983	0.9915	0.82263

**Table 6:** MSD data analysis for PE/NR modified asphalt.

Equation	y=a+b∗x				
Plot	asphaltene	resin	saturate	aromatic	NR
Weight	No Weighting				
Intercept	1.61155 ± 0.11877	1.75603 ± 0.12832	2.74225 ± 0.25882	1.53794 ± 0.05243	2.10403 ± 0.06517
Slope	0.02598 ± 0.00232	0.07596 ± 0.0025	0.14313 ± 0.00505	0.03484 ± 0.00102	0.0455 ± 0.00127
Residual Sum of Squares	0.82214	0.95978	3.90458	0.1602	0.24752
Pearson’s r	0.94514	0.99194	0.99079	0.99359	0.99419
R-Square (COD)	0.89329	0.98395	0.98166	0.98722	0.98842
Adj. R-Square	0.88618	0.98288	0.98044	0.98637	0.98764

## Data Availability

Not applicable.
